# First principles design of two dimensional TiSSe Janus drug delivery system for nitrosourea†

**DOI:** 10.1039/d4ra05119j

**Published:** 2024-10-03

**Authors:** Diwei Shi, Zhengwei Yan, Shiyu Du

**Affiliations:** a School of Naval Architecture and Maritime, Zhejiang Ocean University Zhoushan Zhejiang 316022 People's Republic China shidiwei@zjou.edu.cn; b School of Mechanical Engineering, Nantong Institute of Technology People's Republic China; c Engineering Laboratory of Advanced Energy Materials, Ningbo Institute of Materials Technology and Engineering, Chinese Academy of Sciences Ningbo Zhejiang 315201 People’s Republic of China

## Abstract

In this paper, we delved into the exploration of a novel drug delivery platform for nitrosourea, leveraging a Janus-structured two-dimensional material, TiSSe, as the carrier. Our approach was grounded in a comprehensive application of first-principles computational methods. By evaluating the adsorption energies across a spectrum of potential configurations, we demonstrated the favorable attributes of TiSSe as a carrier for nitrosourea. Our in-depth examination of the electronic structure unveiled intriguing insights. The Janus nature of TiSSe imparts distinct adsorption profiles to nitrosourea molecules at the sulfur (S) and selenium (Se) terminated surfaces. This disparity in electronic properties not only facilitates precise detection but also helps the design of more intriguing two-dimensional materials.

## Abstract 2

We investigated a new drug delivery system for nitrosourea using a two-dimensional carrier with a Janus structure TiSSe through systematic first principles calculations.

## Graphical abstract

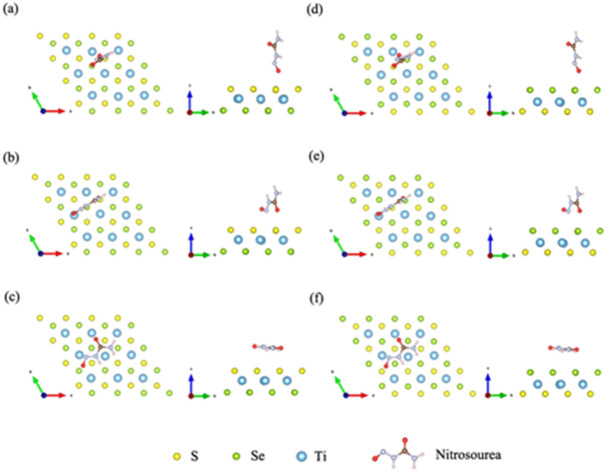

## Introduction

In the interdisciplinary field of nanotechnology and materials science, two-dimensional (2D) materials with Janus configuration have become a hot topic of research due to their unique structural features and functional diversity.^1,2^ These materials integrate two different chemical compositions or surface properties within a single plane, providing a new approach to multifunctional integration and precise control.^3–6^ Especially in the design of drug delivery systems, Janus 2D materials show great potential, enabling efficient drug loading, stable release, and targeted delivery through their distinct surface properties.^2,7^ In addition, Janus 2D materials have also become carriers for material applications in biomedical sensors due to their unique surface characteristics.^8–10^ Furthermore, Janus two-dimensional materials exhibit potential as both emulsifiers and cross-linkers for polymer composites, broadening their applicability to innovative areas like waterproof yet breathable apparel, advanced baby diapers, maternal and infant care products, as well as smart membranes for chemical separation, thereby offering fresh perspectives in these diverse industries.^11–13^

Transition metal dichalcogenides (TMDs), as a class of materials with rich chemical compositions and excellent electronic properties, have been widely applied in the fields of energy storage, catalysis, and sensors.^2,14^ Particularly, when TMDs exist in the form of Janus structures, such as WSSe,^15–17^ MoSSe,^15,18,19^ NbSSe,^20^ and other typical Janus TMDs, their prospects in nano drug delivery systems are particularly eye-catching. Simply put, these Janus-structured TMDs not only provide more chemically active sites but also optimize the interaction between drugs and materials by regulating the properties of different surfaces.^21–24^ Nitrosourea drugs, as an important class of anticancer drugs, are limited in clinical application by stability and side effects.^25–27^ To improve the therapeutic effect of nitrosourea drugs and reduce side effects, the development of a new type of nano drug delivery system is particularly important.

Janus 2D system based on TMDs, with their unique physicochemical properties and tunable surface functions, provide new possibilities for the efficient delivery of nitrosourea drugs. However, there is a lack of research on nitrosourea drug systems using Janus-structured like TiSSe as a carrier. Therefore, this work aims to use first-principles calculations to deeply explore the application of Janus 2D materials based on TMDs in nitrosourea nano drug delivery systems, especially involving properties related to drug adsorption. We will analyze the electronic structure, surface characteristics, and interaction mechanisms of this material with nitrosourea molecules. This study is expected to provide a theoretical basis and guidance for the design of nitrosourea drug nano drug delivery systems, promote the development of new types of efficient, low-toxicity anticancer drug delivery systems, and provide new strategies for cancer treatment.

## Method and computational details

In this paper, all the calculations were processed by code of Vienna *ab initio* simulation package (VASP).^28^ The generalized gradient approximations (GGA) coupled with the Perdew–Burke–Ernzerhof (PBE) is applied as exchange correlation function.^29^ The projector-augmented wave (PAW) method^30,31^ is adopted in the simulation with the employment of VASP, which can precisely determine the electronic valence shell of Ti (3p4s3d), S (3s3p) and Se(4s 4p). 3 × 3 × 1 *k*-mesh in Brillouin zone is sampled for the models using Monkhorst–Pack in the calculation. The van der Waals interaction energies are taken into consideration for the calculation of nitrosourea-adsorption. The specific method used for adsorption in this paper is DFT-D3, which has been widely applied to two-dimensional materials and proven to be reliable.^32–34^ The cutoff energy of the plane wave is set to be 500 eV. Structural relaxation was performed under the condition of the force on per atom less than 0.005 eV Å^−1^.

The zero-point energy (ZPE) has been considered when calculating the Gibbs free energy of the structures containing nitrosourea molecule. The expression formula of ZPE is:1
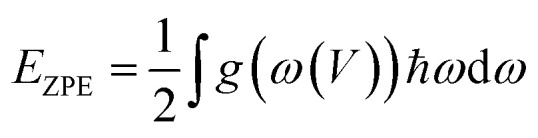
*g*(*ω*(*V*)) represents the phonon density of the states at the given volume, and the finite displacement method was adopted to calculate ZPE with the implement of the PHONOPY software coupling with VASP.

The expression formula of adsorption energy is as follows:

2 *E*_ad_ = *E*_total_ − *E*_bulk_ − *E*_nitrosourea_

*E*_total_ denotes the total Gibbs free energy of adsorption structure, *E*_bulk_ is the Gibbs free energy of Janus Ti_16_S_16_Se_16_ structure, and *E*_nitrosourea_ represents the Gibbs free energy of nitrosourea molecule considering ZPE.

The crystal orbital Hamilton population (COHP) is employed to analyze chemical bonds, which is achieved by LOBSTER.^35^ We use VESTA software for the visualization of the structural drawings in this paper.

## Results and discussion

**Fig. 1:**
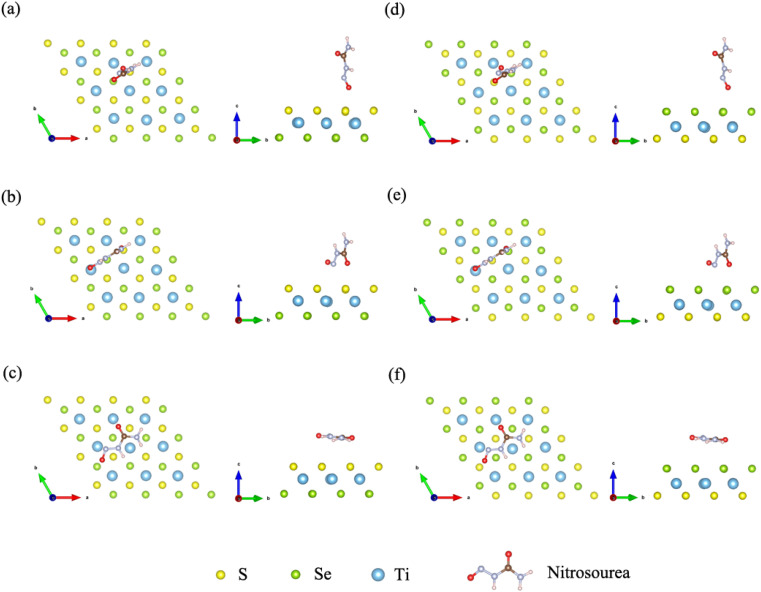
Schematic diagrams for the configurations of the nitrosourea molecule adsorbed on S-layer (a–c) and Se-layer (d–f) of Janus TiSSe.

Janus TiSSe is composed of three layers of Ti, S and Se atoms, showing a sandwich-like structure, of which the outer layers are exposed Se and S atomic layers respectively. In Janus TiSSe, the Ti atoms in the middle layer are arranged in a diamond shape, with the S-atom layer located vertically in the center of the triangle above the Ti-atom layer, and the Se-atom layer positioned vertically on the other side of the Ti-atom layer relative to the S-atom layer. This sandwich-like structure, known as Janus TiSSe, belongs to space group no. 156. A 4 × 4 × 1 Ti_16_S_16_Se_16_ supercell derived from the Janus TiSSe primitive cell are created as the adsorbing bulk, and nitrosourea is adsorbed onto the adsorption layer of S layer or Se layer as adsorbing molecule. Subsequently, we carried out adsorption studies of the nitrosourea molecule at various adsorption sites on the Se and S atomic layer sides of the Janus Ti_16_S_16_Se_16_ bulk structure, considering all possible cases where the nitrosourea molecule is located in vertical, transverse and horizontal positions. Following the establishment of the vacuum layer, we employed the plane-wave method to optimize the structure of the entire system comprising the layer and adsorbed molecules. Hence, a total of three distinct adsorption sites have been identified and are presented in Fig. 1, representing the six optimized adsorption models. Next, we take the adsorption structure c as an example to conduct self-consistent convergence tests. The specific values are shown in the Table S1.† For more complex systems, convergence tests for energy, *k*-points, and cut-off energy have demonstrated the rigor of adopting these parameters.

Next, we further determine the stability of the adsorption structures through the adsorption energy. The drug molecule was geometrically optimized using DFT, and the corresponding data was shown in Table 1. The structural optimization of all adsorption models was conducted within the prescribed accuracy, and furthermore, the energy of the drug molecules was determined after optimization in the process of calculating the adsorption energy. The zero point vibration energy of nitrosourea molecule is calculated to be 1.279 eV, the adsorption energies of the initially stable adsorption structures are listed in Table 1. A negative value of adsorption energy indicates that the adsorption structure is stable, while a positive value may represent a relatively unstable adsorption structure. The adsorption energies of all six CN_3_H_3_O_2_–Ti_16_S_16_Se_16_ structures are negative, thereby confirming the stability of the adsorption systems. In addition, we calculated the contribution of vdW interaction energy to stress in the *z*-axis direction considering the adsorption direction. The specific data is shown in Table S2.† It can be seen that the non-covalent interaction of adsorption structures c and f in the *z*-axis direction has a relatively large impact.

**Table 1:** Adsorption energy of nitrosourea molecules adsorbed on Janus TiSSe

Configurations	Adsorption system (eV)	Nitrosourea (eV)	Bulk (eV)	ZPE (eV)	Adsorption energy (eV)
a	−345.970	−55.110	−290.940	1.279	−1.198
b	−345.932	−55.110	−290.940	1.279	−1.161
c	−346.027	−55.110	−290.940	1.279	−1.256
d	−346.043	−55.110	−290.940	1.279	−1.272
e	−345.884	−55.110	−290.940	1.279	−1.113
f	−345.970	−55.110	−290.940	1.279	−1.119

**Fig. 2:**
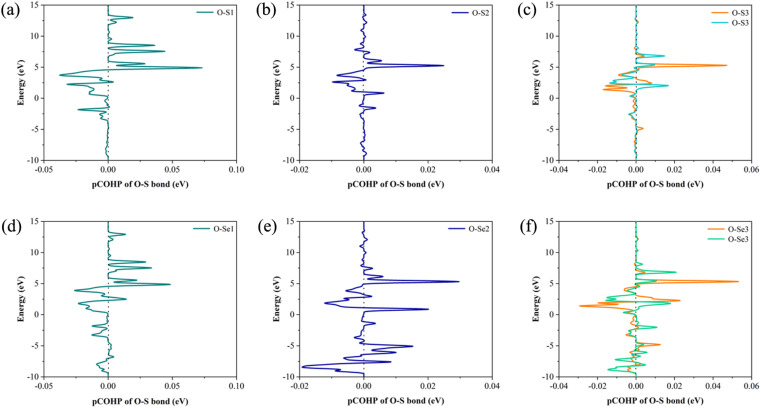
The COHP of O–S and O–Se bonds for the corresponding 6 configurations of the Nitrosourea molecule adsorbed on S-layer (a–c) and Se-layer (d–f) of Janus TiSSe.

In order to explore the effect of bonding on adsorption stability, we further analyzed the bonding during doping and adsorption by calculating COHP. Generally, a positive integral value of COHP indicates bonding orbital between the two atoms, and *vice versa* antibonding. The adsorption bonding is mainly formed by the interaction between the S or Se atom and oxygen atom in nitrosourea molecule, and the specific COHP integral values ICOHP are listed in Table 2. When considering the adsorption stability of nitrosourea molecule on Janus TiSSe, the O–S and O–Se bonds play a pivotal role, existing primarily in bonding state. Notably, the bonding strength of O–Se surpasses that of O–S, indicating that the Se surface of Janus TiSSe favors the loading of nitrosourea molecules. During nitrosourea molecule vertical adsorption, the O–S(Se) bond exhibits the relatively superior binding strength. Conversely, in horizontal adsorption, although more O–S(Se) bonds are established, the binding strength remains unremarkable (Fig. 2).

**Table 2:** The COHP integral values of O–S and O–Se bonds in the adsorption structures

Configurations	Bond	ICOHP
a	O–S	0.0424
b	O–S	0.0336
c	O–S	0.0028
	O–S	0.0023
d	O–Se	0.0492
e	O–Se	0.0489
f	O–Se	0.0084
	O–Se	0.0043

**Fig. 3:**
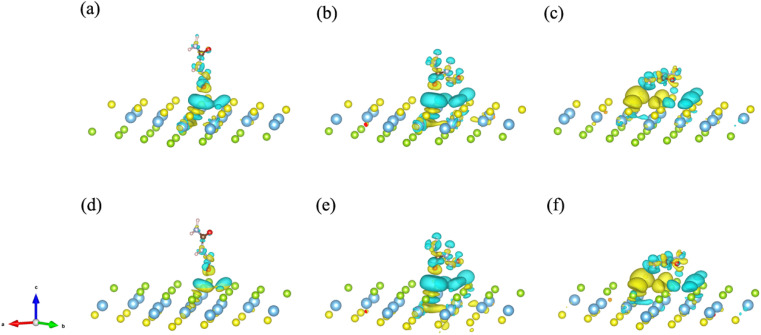
The electron charge density difference (CDD) for the systems of nitrosourea molecule adsorbed on S-layer (a–c) and Se-layer (d–f) of Janus TiSSe.

To further explore and verify charge transfer in layered materials during the adsorption processes, the electronic properties of the Janus TiSSe adsorbing nitrosourea were analyzed, including the electron charge density difference (CDD) and partial density of states (PDOS). The schematic diagrams of electron charge density difference for the six structures are shown in Fig. 3. Yellow areas show charge accumulation, while cyan areas indicate charge depletion. It can be found that during the adsorption processes, the bulk materials exhibit obvious charge transfer phenomenon, which reflects on the strong interaction between the S or Se atoms and the oxygen atom in nitrosourea closest to the adsorption layer, which is also supported by the analysis of the above-mentioned ICOHP.

From Fig. 3, it is evident that upon the adsorption of nitrosourea molecules onto the S layer of Janus TiSSe, there is a pronounced charge accumulation surrounding the O–S bond. Specifically, the S atom layer experiences a loss of charge, which accumulates around the O atoms. This observation indicates that the S layer of Janus TiSSe possesses a substantial adsorption capacity for nitrosourea molecules positioned at three distinct sites. In addition, the nitrosourea molecule exhibits a preference for adsorbing horizontally onto the S layer of Janus TiSSe, forming bonds with multiple S atoms. The augmentation in the number of interaction sites substantially diminishes the adsorption energy barrier, thereby providing a compelling justification for the observed minimal adsorption energy exhibited by horizontally oriented nitrosourea molecules. With regard to the Se layer of Janus TiSSe, the pattern of charge transfer indicates that nitrosourea molecules can achieve stable adsorption on this layer through all three possible adsorption modes. Notably, the multi-site vertical and horizontal adsorption configurations exhibit a significantly more pronounced charge transfer phenomenon in comparison to the single O-site adsorption mode. It is noteworthy that the bonding between horizontally adsorbed nitrosourea molecules and the S atoms displays a distinctive pattern of concurrent charge accumulation and depletion, which is likely attributed to the uneven charge distribution inherent to the nitrosourea molecules. To further elucidate the bonding mechanisms between nitrosourea and the S and Se layers of Janus TiSSe, we have undertaken detailed calculations of the density of states.

**Fig. 4:**
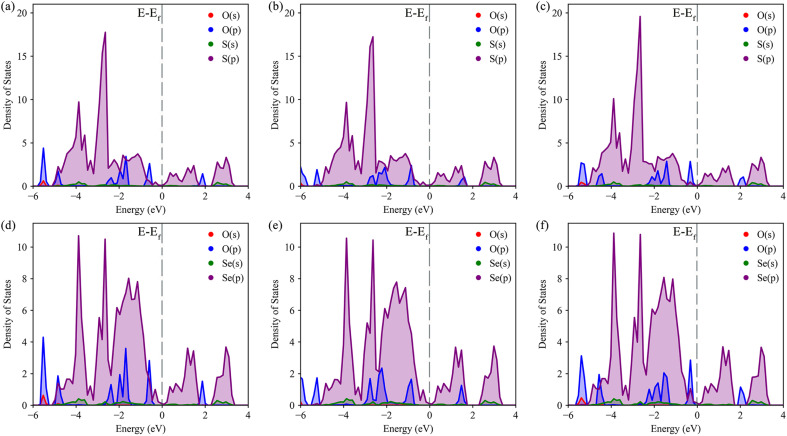
The density of states (DOS) for the systems of nitrosourea molecule adsorbed on S-layer (a–c) and Se-layer (d–f) of Janus TiSSe.

The density of states (DOS) was calculated to further understand the interaction between O–S and O–Se, as well as the specific transfer of orbital charge. The DOS diagrams for all six structures are shown in Fig. 4. Initially, we focused on the DOS of three distinct S-layer adsorption configurations on the Janus TiSSe surface. As evident from Fig. 4a–c, the primary contribution to the Fermi energy level stems predominantly from the p-orbitals of the S element, with a notable contribution also from the p-orbitals of the oxygen element. Intriguingly, the variations in the p-orbitals of the oxygen atom, arising from changes in the adsorption configuration, directly correlate with alterations in the adsorption energy. Besides, for the three Se-layer adsorption structures in Fig. 4d–f, the p-orbitals of the Se element emerge as the dominant contributor to the Fermi energy level. Within the energy range of −2 to 2 eV near the Fermi surface, a distinct bonding pattern emerges between the p-orbitals of the O atom and the p-orbitals of the Se element. Based on the observed changes in differential charge density and density of states (DOS), there is a clear indication of charge transfer between the adsorbed molecules and the layer, potentially signifying the formation of weak molecular or ionic bonds. Analogous to the DOS analysis of S-layer adsorption, the different adsorption configurations lead to subtle variations in the orbital electron distributions of the O atom, which ultimately influence the adsorption behavior of the nitrosourea molecule. Overall, owing to the Janus structure of TiSSe, the nitrosourea molecule exhibits distinct adsorption properties at the S- and Se-ends, facilitating its specific detection.

Among various drug delivery platforms, the anisotropic nanoparticles of Janus (JNPs) exhibit distinct advantages in drug delivery despite ongoing challenges related to their physical stability in biological media and biosafety aspects.^36^ The homogenous nanoparticles restrict co-delivery to either hydrophobic or hydrophilic drugs, whereas complex JNPs uniquely encapsulate both hydrophobic and hydrophilic drugs, enabling simultaneous release for synergistic effects.^37,38^ Their high surface-to-volume ratio optimizes targeting ligands and can be compartmentalized for imaging agents. On the other hand, discovering an efficient prep-purification method for large-scale JNP production is challenging.^39^ Furthermore, JNPs featuring diverse surface chemistries, though offering richer opportunities for unique feature integration, complicate interactions compared to uniform nanoparticles. Notably, insufficient understanding exists regarding the cellular entry mechanisms of JNPs with both sensing and targeting compartments, particularly whether they penetrate cells akin to uniform particles solely equipped with sensing or targeting capabilities.^40^ While this article presents a computational study rooted in density functional theory, the methodology herein primarily focuses on investigating the ground-state properties of the system. For other, more macroscopic properties such as plasticity, alternative simulation methodologies are necessitated for comprehensive exploration.

## Conclusion

In this study, we investigated a new drug delivery system for nitrosourea using a two-dimensional carrier with a Janus structure TiSSe through systematic first principles calculations. By calculating the adsorption energies of several different adsorption configurations, it is shown that TiSSe is a promising carrier for nitrosourea. Furthermore, through detailed analysis of the electronic structure, the results indicate that due to the Janus structure of TiSSe, nitrosourea molecules exhibit different adsorption characteristics at the S- and Se-terminations. This difference in electronic characteristics is helpful for its specific detection, and indicates that TiSSe, as a Janus carrier, has potential applications for sensors and targeting materials.

## Data availability

Data will be made available on request.

## Conflicts of interest

The authors declare that they have no known competing financial interests or personal relationships that could have potentially influenced the findings presented in this paper.

## Supplementary Material

RA-014-D4RA05119J-s001
