# Supplementary material for: First principles design of two dimensional TiSSe Janus drug delivery system for nitrosourea
Source: RSC Adv. 2024 Oct 3;14(43):31433–8. doi: 10.1039/d4ra05119j (PMC11447516; doi:10.1039/d4ra05119j)
Supplement: RA-014-D4RA05119J-s001 [file RA-014-D4RA05119J-s001.pdf]

Supplementary Information (SI) for RSC Advances.
This journal is © The Royal Society of Chemistry 2024

First principles design of two dimensional TiSSe Janus drug delivery system for Nitrosourea

Diwei Shi[a]*, Zhengwei Yan[b]

a. School of Naval Architecture and Maritime, Zhejiang Ocean University, Zhoushan, Zhejiang 316022, People's Republic China

b. School of Mechanical Engineering, Nantong Institute of Technology

*Correspondence to: shidiwei@zjou.edu.cn

Table S1. Convergence tests for different energies, k-points, and cut-off energies

system energy (eV)	cutoff energy 300 eV	cutoff energy 500 eV	cutoff energy 550 eV	cutoff energy 600 eV
k-mesh 1×1×1	-345.145	-345.023	-344.985	-345.010
k-mesh 3×3×1	-346.096	-346.027	-346.044	-346.073
k-mesh 5×5×1	-346.197	-346.141	-346.145	-346.174

Table S2. The contribution of vdW interaction energy to stress in the z-axis direction.

configuration	vdW on Z direction (eV)
a	-0.782
b	-1.297
c	-2.291
d	-0.851
e	-1.276
f	-2.232
